# Potential Psychotropic and COVID-19 Drug Interactions: A Comparison of Integrated Evidence From Six Database Programs

**DOI:** 10.7759/cureus.20319

**Published:** 2021-12-10

**Authors:** Javedh Shareef, Sathvik Belagodu Sridhar, Sabin Thomas, Atiqulla Shariff, Sriharsha Chalasani

**Affiliations:** 1 Clinical Pharmacy, Ras Al Khaimah Medical and Health Sciences University (RAKMHSU), Ras Al Khaimah, ARE; 2 Clinical Pharmacy & Pharmacology, RAK College of Pharmaceutical Sciences, Ras Al Khaimah Medical and Health Sciences University (RAKMHSU), Ras Al Khaimah, ARE; 3 School of Pharmacy/Pharmacy Practice, College of Pharmacy & Nursing, University of Nizwa, Nizwa, OMN; 4 Pharmacy Practice, Jagadguru Sri Shivarathreeshwara (JSS) College of Pharmacy, Mysuru, IND

**Keywords:** covid-19 drug, mental disorders, psychotropic drugs, drug interactions, patient care

## Abstract

Background

Drug interactions are a significant issue in mental illnesses and coronavirus disease 2019 (COVID-19) infections. Inconsistency in drug interaction resources makes prescribing challenging for healthcare professionals. To assess the scope, completeness, and consistency of drug-drug interactions (DDIs) between psychotropic and COVID-19 medications in six specific drug information (DI) databases.

Methodology

For the comparison, six DI resources were used: Portable Electronic Physician Information Database, Micromedex®, Medscape.com, UpToDate®, Drugs.com drug interaction checker, and WebMD.com drug interaction checker. Using the Statistical Package for the Social Sciences (SPSS) software version 27 (IBM Corp., Armonk, NY), the gathered data were examined for scope, completeness, and consistency.

Results

Scope scores were higher for PEPID© than all the other resources (p < 0.001) for each comparison. PEPID© had better overall completeness scores (median 5, Interquartile range [IQR] 5 to 5; p<0.05 for each comparison), except for Drugs.com (p < 0.05 for each comparison), and were more remarkable for Micromedex® (median 5, IQR 5 to 5). The Fleiss kappa scores among the six different DI sources were poor (k < 0.20, p < 0.05) for the category of information related to clinical effects and level of documentation, moderate agreement (k = 0.4 - 0.6, p < 0.05) for the severity and course of action of DDIs, and fair agreement (k = 0.4 - 0.6, p < 0.05) for mechanism.

Conclusion

A comprehensive, accurate information among DI resources is essential for healthcare professionals that will significantly impact patient care in the clinical practice. Banking on high-quality resources will help healthcare professionals to make an informed decision while prescribing to avoid inappropriate combinations that can adversely affect patient outcomes.

## Introduction

Coronavirus disease 2019 (COVID-19) continue to be pandemic affecting millions of people worldwide, leading to significant morbidity and mortality. The fast and broad spread of COVID-19 has become a worldwide health risk factor, responsible for various clinical presentations ranging from no signs of illness to severe respiratory symptoms linked with multi-organ failure. People over the age of 65 who have a history of chronic medical problems such as cardiovascular disease, diabetes, respiratory diseases, and cancer are at a higher risk of contracting COVID-19 [1].

Given the possibility that patients with psychiatric disorders are more likely to be infected with COVID-19, it has been reported that up to 20%-30% of patients infected with COVID-19 will have or develop delirium or other psychiatric illness, with a higher number in chronic disease patients either during their hospitalization or as part of treatment-related adverse effects. These psychological problems might cause reduced immunity requiring advanced disease prevention and management strategies. For these reasons, individuals infected with COVID-19 may need pharmacotherapy aimed at psychiatric illness or may need to continue taking psychiatric medicines for pre-existing psychiatric problems while infected with COVID-19 [2,3].

At present, no pharmacological treatments have been approved by the WHO or proven safe and effective for managing COVID-19 infection. However, a significant number of experimental medicines are being used on an off-label basis on an as-needed basis or as part of existing clinical procedures for treating COVID-19 infection [4].

Because of the many concerns about the safety and effectiveness of these medicines, prescribing antipsychotic medications to COVID-19 patients will be increasingly difficult. In addition, most antipsychotic medications and fewer COVID-19 medications are metabolized via cytochrome P450 enzymes, increasing the risk of drug-drug interactions [5].

Drug interactions may vary from those that are not clinically significant to those that can cause life-threatening (or) irreversible harm, affecting therapeutic response and worsening therapeutic outcomes [6]. In addition, age-related decline in drug metabolism, an increase in the incidence of comorbidities such as hepatic & renal impairment, and varying drug plasma concentrations complicate the use of medications and increase the sensitivity to drug interactions [7]. As a result, drug interactions prolong hospital stay, increase healthcare expenditure, and worsen patient outcomes in inpatient and outpatient settings [8].

Several drug information resources/databases providing information on various aspects of medications, including drug interactions, are available to enhance patient safety and provide a high quality of medical care. These are printed resources with ready-reference information, such as the British National Formulary, Stockley's Drug Interactions, the American Hospital Formulary Service, and so on. In addition, there are subscription-based databases such as Micromedex® PEPID© (Portable Electronic Physician Information Database) & UpToDate and freely accessible databases (Medscape drug reference, Drugs.com, Epocrates.com, and WebMD.com) resources that facilitate evidence-based practice for healthcare professionals. However, the ultimate choice on which database to seek information from, purchase, or subscribe to is likely influenced by subjective factors such as the convenience of use, familiarity, or quick access during professional practice. [9].

Research comparing clinical decision support tools for drug information utilizing online drug information databases found that subscription databases outperformed freely available online databases in their ability to answer queries across various categories [10].

Few observations have identified significant variability among the resources related to drug interactions such as severity, mechanism of action, clinical effects, level of documentation, and management. Furthermore, it creates uncertainty for healthcare professionals in prescribing medications, especially those at risk of developing drug interactions [11]. Moreover, healthcare professionals should be aware of the quality and extent of consistency of different resources that are commonly available, which will be highly advantageous in their clinical practice. Although investigations on antipsychotic drug interactions with COVID-19 medicine have been reported, studies assessing drug interaction resources are sparse. As a result, the current research assesses the consistency, completeness, and scope of drug-drug interactions involving psychiatric medicines and COVID-19 medications from six database resources for moderate-severe/contraindicated/life-threatening possible drug-drug interactions.

## Materials and methods

A systematic comparative study was carried out. Six drug information resources widely used by the healthcare professionals in the United Arab Emirates were chosen for comparison in order to investigate possible drug-drug interactions between antipsychotic medicines and COVID-19 medications. These include subscription databases, namely Micromedex® [12], PEPID© [13], UpToDate® [14], and freely accessible online databases such as Medscape.com, Drugs.com, and WebMD’s drug interaction checker [15-17]. In addition, information on repurposed COVID-19 medications or under investigation agents for COVID-19 treatment were compiled from several guidelines and by doing a literature search in the PubMed, Google Scholar, Scopus, and Web of Science databases, which are all up to date until May 2021 [18,19].

The research covered the various categories of psychotropic medicines outlined by the Anatomical Therapeutic Chemical (ATC) classification system for treating various psychiatric illnesses such as anxiolytics, antidepressants, antipsychotics, and mood stabilizers. These selected psychotropic medications were added to the 'drug to check' list in the interaction tool of the different drug-interaction database and free online resources, along with the COVID-19 drugs, to identify the initial moderate-severe/contraindicated/life-threatening potential drug interactions between psychotropic drugs and COVID-19 medications (Table 1).

**Table 1:** List of psychotropic drugs and COVID-19 medications included for the drug interactions. SSRIs: selective serotonin reuptake inhibitors; SNRIs: serotonin-norepinephrine reuptake inhibitors; TCAs: tricyclic antidepressants; MAOIs: monoamine oxidase inhibitors.

COVID-19 medications	Psychotropic drugs
Hydroxychloroquine	Antidepressants
Lopinavir	SSRIs
Ritonavir	Fluvoxamine
Azithromycin	Fluoxetine
Dexamethasone	Sertraline
Ivermectin	Citalopram
Remdesivir	Escitalopram
Interferon	Paroxetine
Tocilizumab	SNRIs
	Duloxetine
	Venlafaxine
	TCAs
	Amitriptyline
	Nortriptyline
	Imipramine
	Clomipramine
	Doxepin
	MAOIs
	Isocarboxazid
	Phenelzine
	Tranylcypromine
	Others
	Buproprion
	Mirtazapine
	Trazodone
	Vilazodone
	Vortioxetine
	Antipsychotics
	Chlorpromazine
	Haloperidol
	Trifluoperazine
	Quetiapine
	Risperidone
	Olanzapine
	Aripiprazole
	Ziprasidone
	Clozapine
	Mood Stabilizers
	Carbamazepine
	Valproic acid
	Lamotrigine
	Lithium
	Anxiolytics
	Diazepam
	Midazolam
	Alprazolam

The research team selected and prepared a list of potential drug interaction pairs between psychotropic drugs and COVID-19 medications and was sent to four expert reviewers. Each drug interaction pair containing at least one psychotropic drug and a COVID-19 medication were included in the study. Drug interactions among the psychotropic drugs and also between the COVID-19 medications were not considered for the study. The expert reviewers included one psychiatrist, one senior clinical pharmacist, and two drug information pharmacists.

The primary purpose of the expert review was to ensure that all pairs of drugs selected are relevant and there is a possible chance of potential drug-drug interaction likely to occur and to identify any additional drug pairs that need to be included in the list. Based on the experts' review, the final sample yielding 181 potential drug interaction pairs of moderate-severe/ life-threatening that require an intervention was considered for the study. Therefore, we have excluded the drug interaction pairs rated severity level as ‘minor’ or ‘no action required. Furthermore, the research did not assess possible drug interactions of psychotropic and COVID-19 medications with alcohol, food, smoking, nutritional supplements, or illicit substances.

Using a common computerized data collection form, three independent study investigators gathered data from each resource. Any disagreements between the investigators were settled by consensus. Collected data regarding the frequency, severity, type and documentation of drug-drug interactions were entered into an excel spreadsheet for further analysis.

The three vital assessed endpoints utilized to assess the research objectives were scope, completeness, and consistency. The scope is defined as “Presence of interaction as an entry in the resource, computed as a percentage of interactions that had an entry for each resource.” While Completeness was defined as “resource containing clear and precise information addressing each of the extracted components namely mechanism (pharmacodynamics/pharmacokinetic description how the interaction occurs), severity (seriousness of the of the interaction if it occurs), clinical effects (signs/symptoms and patient outcomes resulting from the interaction if it occurs), level of documentation (level of certainty supporting the likelihood of the interaction) and course of action (suggested necessary actions to ease the interaction).” The completeness score was computed as “a percentage of interaction with an entry describing each element individually” [20,21].

Because each drug information resource uses a different scale to evaluate the severity of interactions, we categorized them as moderate, significant, or contraindicated based on the description given in each resource to standardize the risk outcome levels of interactions (Table 2).

**Table 2:** Standardization of risk outcome of drug interactions. Up To Date: C = Monitor therapy, D = Consider therapy modification, X = Avoid combination. Portable Electronic Physician Information Database (PEPID©) : 3 = Moderate, 4 = Significant, 5 = Life Threatening.

Drug interaction severity	PEPID©	Micromedex®	UpToDate	Drugs.com	Medscape.com	WebMD.com
Moderate	3	Moderate	C	Moderate	Monitor closely	Monitor closely
Major/Contraindicated	4,5	Major, Contraindicated	D,X	Major	Serious	Serious, Don’t use together

The overall completeness score was determined by assigning one point to each component and adding the scores of the five components to give a value ranging from 0 to 5 for each resource interaction. For example, suppose an interaction entry provided clear, precise information addressing all the five components. In that case, it will have a total score of five, whereas if an interaction provided information describing the only mechanism, severity, and course of action and does not address clinical effects and level of documentation, it would receive only three out of five.

To determine the consistency among the different resources, the investigator reviewed the data collected for each interaction. Then, the consistency scores were calculated using the percentage of interaction in each resource compared to the majority of the resources.

The indicators utilized to evaluate the consistency across six resources were content for severity and course of action. The severity level for each interaction was classified as minor, moderate, major, and severe/contraindicated in each resource. Similarly, the course of action suggestions were classified as no action required, monitoring, modifying the dosage, and preventing concurrent use or combination. As a result, the scores for both components were computed as the percentage of ratings similar to the majority of the results from the resources. Since we have not included interaction rated as severity level ‘minor’ in our study, it would not be considered consistent.

Data analysis

The scope, completeness, and consistency scores were described using descriptive statistics like numbers and percentages. The data were analyzed using the Statistical Package for the Social Sciences (SPSS) software version 27 (IBM Corp., Armonk, NY).

The median and interquartile ranges were used to calculate the overall completeness of the scores. Because all resources were assessed in pairs for the same interactions, the scope scores were compared using the McNemar test, and the overall completeness ratings were compared using the Wilcoxon-signed-rank test.

Because the consistency ratings were calculated using two specific measures (severity and course of action). In a few instances where only two or fewer resources contained the entry with the information, the statistical significance of the findings was not evaluated. Finally, the concordance of the six references was determined by Fleiss kappa (k) co-efficient to evaluate the reliability of agreement between the investigators and among the different drug interaction resources in assigning categorical ratings. 

Landis and Koch's criteria are adapted to calculate the degree of agreement of the Fleiss kappa value: A score of 0.2 indicates weak agreement, a score of 0.21-0.40 shows fair agreement, a score of 0.41-0.60 indicates moderate agreement, a score of 0.61- 0.80 indicates substantial agreement, and a score of 0.81-1.00 indicates almost perfect agreement [22]. In addition, a p‐value is calculated for each kappa with a p-value of <0.05, indicating statistical significance.

## Results

The investigators identified 181 potential drug interactions with moderate-severe clinical significance, and no other interactions were included following the subject experts' review. So the final sample was confirmed with 181 drug interactions for analysis.

Scope scores

PEPID© had the highest scope score of 79% (143 out of 181), followed by Drugs.com at 63.5% (115 out of 181) and Medscape at 52.4% (95 out of 181). Thus, scope scores were significantly higher (p<0.001 for each comparison) for PEPID© as compared to all the other resources (Table 3).

**Table 3:** Scope score for the drug interaction resources.

Drug Information resource	Total number of drug interactions (n=181)	
	n	%
PEPID©	143	79
Micromedex®	85	46.9
UpToDate	71	39.2
Medscape.com	95	52.48
Drugs.com	115	63.53
WebMD	81	44.75

Completeness scores

The completeness scores for the drug interaction studies were analyzed in the five-specific components describing each of the extracted items. The completeness score for the mechanism ranged from 64.19% (WebMD) to 100% (PEPID©, Drugs.com) and for clinical effects ranged from 20% (Drugs.com) to 95.2% (Micromedex®). Almost all the resources provided 100% (PEPID, Micromedex®, UpToDate, Drugs. Com, WebMD) completeness score for mechanism except Medscape (98.94%). The course of action was 100% for PEPID©, Micromedex®, Drugs.com, and WebMD, followed by 98.94% for Medscape.com and 92.95% for Up to Date. 

Overall completeness ratings varied from 3 (IQR 3 to 3, WebMD) to 5 (IQR 5 to 5, PEPID, Micromedex®). The completeness tier assessment contributed to the creation of five tiers, the first of which is tier 1 (PEPID; median 5, IQR 5 to 5; p <0.05 against all remaining resources for each comparison) and except for Drugs.com [p>0.05], Micromedex® (median 5, IQR 5 to 5; p0.05 against all other resources for each comparison); tier 2 (UpToDate; median 4, IQR 4-5; p < 0.05 against all other resources for each comparison except Medscape.com (p>0.05).

Whereas in tier 3 (Drugs.com; median 4, IQR 3 to 4; p: 0.05 against all other resources for each comparison), tier 4 (Medscape.com, median 3, IQR 3 to 4; p < 0.05 versus all other resources), and tier 5 (Medscape.com, median 3, IQR 3 to 4; p < 0.05 versus all other resources) (WebMD; median 3, IQR 3 to 3) (Table 4).

**Table 4:** Completeness elements and overall completeness scores for the drug pairs with entries. PEPID© = Portable Electronic Physician Information Database.

Resource	Mechanism	Clinical effects	Severity	Level of documentation	Course of action	Overall completeness	
	n (%)	n (%)	n (%)	n (%)	n (%)	Median	IQR
PEPID© (n =143)	143 (100)	114 (79.7)	143 (100)	139 (97.2)	143 (100)	5	5 to 5
Micromedex® (n = 85)	83 (97.6)	81 (95.2)	85 (100)	85 (100)	85 (100)	5	5 to 5
Up to Date ( n = 71)	68 (95.7)	26 (36.6)	71 (100)	71 (100)	66 (92.9)	4	4 to 5
Drugs.com ( n = 115)	115 (100)	23 (20.0)	115 (100)	45 (39.1)	115 (100)	4	3 to 4
Medscape.com (n = 95)	71 (74.7)	36 (37.8)	94 (98.9)	16 (16.8)	94 (98.9)	3	3 to 4
WebMD.com (n = 81)	52 (64.1)	21 (25.9)	81 (100)	16 (19.7)	81 (100)	3	3 to 3

Consistency scores

Consistency scores for severity ranged from 51.42% (UpToDate ) to 80.4% (Micromedex®), and for the course of action, the scores ranged from 46.26% (WebMD) to 66.23% (Micromedex®) (Table 5).

**Table 5:** Consistency scores by various drug interaction resources. PEPID© = Portable Electronic Physician Information Database.

Resource	Severity		Course of action	
	Score	Percentage	Score	Percentage
PEPID©	72/119	60.5	58/121	47.9
Micromedex®	66/82	80.4	51/77	66.2
Up to Date	36/70	51.4	44/70	62.8
Drugs.com	57/75	76.0	44/92	47.8
Medscape.com	77/102	75.4	42/81	51.8
WebMD	55/69	79.7	31/67	46.2

Inter-source reliability analysis

An inter-source reliability study utilized the kappa coefficient to assess the concordance among the various components of the drug interactions resources. It is also used to evaluate the consistency of information across investigators and various drug interaction resources. For example, we perceived a lack of consensus (k value <0.2) among the drug interaction resources and investigators concerning the components‘ clinical effects’ and ‘level of documentation. However, for the information related to the component ‘severity’ and ‘course of action,’ it was identified that there is a ‘moderate’ agreement between the investigators and different drug interaction resources, and for the component ‘mechanism,’ it was decided that this was a fair degree of agreement. In addition, the Fleiss kappa for the overall completeness inter-rater agreement was found to be ‘fair’ (Table 6).

**Table 6:** Inter-reliability analysis for the concordance among the drug interaction resources studied. *p-value <0.05 is statistically significant. ^†^k <0.2 signifies poor agreement.

Components of the drug-drug interactions	Value of k^†^	95% Confidence Interval	p-value*	Strength of agreement
Clinical effects	0.124	0.086 – 0.162	0.001	Poor
Severity	0.483	0.441 – 0.526	0.001	Moderate
Mechanism	0.276	0.233 – 0.318	0.001	Fair
Level of documentation	0.187	0.150 – 0.225	0.009	Poor
Course of action	0.462	0.419 – 0.504	0.001	Moderate
Overall completeness	0.270	0.248 – 0.293	0.001	Fair

## Discussion

Available studies have identified that antipsychotic medications can interact with COVID-19 drugs mainly by pharmacodynamics and pharmacokinetic drug interactions leading to alteration of plasma drug concentrations resulting in a negative therapeutic outcome or increased risk of toxicity [23,24]. As a result, early detection of these interactions and timely interventions will aid in the optimization of medication therapy in the management of psychiatric disorders in COVID-19 patients [25].

Drug-drug interaction databases are vital in assisting physicians while prescribing medications in clinical practice to optimize pharmacotherapy and ensure patient safety. The present study evaluated moderate-severe/contraindicated/life-threatening potential drug-drug interactions from six database resources: three points of care databases (PEPID©, Micromedex®, UpToDate) and three online drug interaction checkers (Drugs.com, Medscape.com, and WebMD).

The research examined the various drug interaction resources for the scope, completeness, and consistency of information linked to potential drug interactions of moderate-severe/life-threatening importance between COVID-19 medicines and psychotropic drugs. The research discovered that the scope of accessible information was limited, with no resources accounting for more than 80% of the interaction sample. Among the drug interaction resources reviewed, PEPID© had most of the studied drug pair’s entry with the highest scoring references in terms of scope score (79%), followed by Drugs.com and Medscape.com with 63.5% and 52.4%, respectively. A study conducted by Beckett RD et al. evaluating DI resources for drug-ethanol and drug tobacco interactions revealed that Lexi comps (84.9%) and clinical pharmacology (83%) had the highest scope score in comparison to our study where PEPID (79%) and Drugs.com (63.5%) had the highest scope score [20]. However, research performed by Shariff A et al. assessing the consistency of information regarding drug-drug interaction across different drug information databases revealed that PEPID and UpToDate had the majority of drug pair's entry with the most excellent scope core (100%) [26]. However, the comparative studies have used different classes of drug combinations compared to our study. 

Marcath LA found that DI resources Lexi comp and Drugs.com were the top-ranking subscription and free online tools analyzed in their research when evaluating the various instruments for screening drug interaction of oral oncolytics [27]. However, no drug information resource enumerates all of the interacting drug combinations in our research among the 181 drug interactions found from six distinct databases. For example, only 11.04 % of the similar drug interaction pairs were identified in all the six resources, and 40.33% of drug interactions were presented only in one or two resources. 

Even though the subscription database (PEPID©) had the highest score for the drug pair entry with resource, few interactions Duloxetine + Ritonavir, Venlafaxine + Lopinavir, Lithium + Hydroxychloroquine, Clozapine + Hydroxychloroquine, Valproate + Remdesivir, Aripiprazole + Dexamethasone had no entry into any of the subscription tools but was available in the free online database resources. Additionally, PEPID© and Micromedex® scored higher overall completeness than UpToDate, Medscape, Drugs.com, and WebMD. Though the study's primary goal was not to define the individual components of completeness, the findings indicated that PEPID, Micromedex®, UpToDate, and Drugs.com might be utilized as resources of choice for evaluating the mechanism of action of an interaction. At the same time, Micromedex® and PEPID© would be more beneficial for identifying the clinical effects.

All the resources reported the severity rating of the interaction and recommended a course of action for the interaction sample. The level of documentation was best narrated in Micromedex®, UpToDate, and PEPID© compared to drugs.com, Medscape.com, and WebMD. A study carried out by Patel et al. evaluating the different resources for analyzing drug interactions showed that Lexicomp interactions and Micromedex® and Stockley's drug interaction were the resources of choice for analyzing the different components of completeness related to drug interactions [21]. Furthermore, this shows that discrepancy exists for the information related to drug interactions among the resources, and healthcare professionals need to be aware that this may change over time as and when the resources get updated [28].

Micromedex® and WebMD ranked best in terms of consistency of severity ratings, while Micromedex® and UpToDate were most suitable for a suggested course of action. These findings echoed previously published results carried out by Patel et al. and Shariff A et al., which showed that Micromedex® ranked the highest consistency score for severity ratings compared to other DI resources [21,26]. In addition, Beckdett et al. found that clinical pharmacology and Lexicomp online had the greatest severity rankings, while drug interaction facts and Micromedex® had the highest recommended course of action rankings [29].

Information on severity about the interaction between two drugs is an essential factor for the healthcare professional in deciding on the clinical use of these combinations in inpatient drug therapy. Usually, interactions rated as mild to moderate and with no action required can be used depending on the patient's clinical conditions and all necessary precautions. However, if the severity of the interaction is rated as major/ serious / contraindicated, then the drug pair must be avoided or should not use together.

We have noticed that there is a discrepancy in information related to severity among the DI resources. We have identified only four pairs of drug interactions of severity inconsistency among all the resources. Among the four-drug pairs, three midazolam + Lopinavir/Ritonavir, Quetiapine + Lopinavir/Ritonavir, Carbamazepine + Ritonavir/Lopinavir were rated as severe /major among all the six resources, and drug pair Olanzapine + Ritonavir were categorized as moderate among all the resources. Surprisingly Olanzapine + ritonavir was categorized as moderate in four resources and no interaction in two resources. In parallel, many of the interaction drug pairs rated as severe/major/life-threatening in most of the resources were rated as moderate/monitored closely in other resources and vice versa.

Disagreement was also noted in assessing the consistency concerning the course of action. Only nine drug interaction combinations had a similar course of action across all resources. The differences varied from PEPID and Drugs.com recommending caution and careful monitoring for the remaining medication combinations to alternative drugs. In contrast, Micromedex® suggests avoiding the combination or decreasing the dose and UpToDate recommends that no action is required for the majority of the patients or consider increased monitoring for signs and symptoms. The course of action provided by Medscape.com suggests using the drug pair with caution/monitor, and the WebMD advises significant interaction possible and requires the monitoring by the doctor. This heterogeneity creates uncertainty among healthcare professionals in deciding whether to use such combinations in patients who need them when there are no other alternatives.

Comprehensive information among the resources is vital as accurate and consistent information helps clinicians prescribe medications to avoid harmful drug interactions. Fleiss kappa measure of interrater reliability is a method that measures the agreement between the raters, and we have used it for examining the agreement among the resources. We have found that the inter-reliability scores of the DI resources for the various components of drug interactions differ in the strength of agreement as assessed by the Fleiss kappa (K) score. For example, the Fleiss kappa coefficient for the category ‘clinical effects ‘and ‘level of documentation of the drug interaction was less than 0.2, indicating poor agreement. Similarly, the kappa coefficient for the category ‘severity’ and ‘course of action was between 0.41-0.6, which shows a moderate degree of agreement, and for the category ‘mechanism,’ the strength of agreement was found to be fair among the different drug interaction resources studied. A study carried out by Monteith et al. comparing the potential psychiatric drug interactions with six database programs identified that the overall interrater Fleiss kappa agreement was found to be fair. In addition, the agreement was substantial and fair for the drug combination categorized as severe and major [28]. Another study carried out by Monteith et al., analyzing the potential drug interactions with drugs used for bipolar disorder by comparison with six drug interaction database programs, documented a lack of consensus across drug interaction database programs for potential psychiatric drug interactions [30].

Above all, none of the resources has stood out with the highest score across all the three assessments, namely scope, completeness, and consistency of the drug interaction sample. Furthermore, this highlights the need to refer to more than one drug interaction resource in clinical practice, as doctors and other healthcare staff depend on similar resources at the time of patient treatment. The authors' view was well supported by Montieth et al. with the similar findings of inconsistency among the database programs for interacting psychiatric drug pairs and suggesting the need to refer multiple database programs by the psychiatrist based on the clinical decision and practicing experience [22,28,30]. Each resource had different strengths and limits in identifying and categorizing drug interactions, and the findings of this research will help healthcare professionals and users depend on high-quality resources for drug interactions. The psychiatrists need to be aware of the inconsistency about the drug interactions resources that may change over time while using the drug pairs safely and effectively without causing any patient harm.

The strength of our study includes the use of subscription-based and freely accessible online-based six different database programs to assess the potential drug interactions between psychotropic and COVID-19 medications for the scope, completeness, and internal consistency of the information. Previous studies compared the database programs for the drug interactions between psychiatric drugs and COVID-19 medications by using pharmacokinetic and pharmacodynamics mechanisms via the cytochrome P450 enzyme system and warranted careful patient monitoring and management [5,22,24].

There were a few limitations to our study. Drug-drug interaction database programs can assist physicians while prescribing medications in clinical practice, but their disagreement limits their use. Currently, data available regarding the drug interaction of certain psychotropic drugs with COVID-19 medications is limited. More scientific findings from observational studies and clinical trials are needed to investigate the nature, effects, and treatment of COVID-19 and psychiatric prescription drug interactions. Another limitation of the study was that only six database resources available as a subscription and free online were evaluated, and we have not included any textbook resources.

Furthermore, the information related to a few drugs was not covered in the textbook resources as it gets their data updated at different time intervals. For example, Stockley's drug interaction was revised and updated every two years, while British National Formulary is revised and updated every six months. This makes it practically impossible to compare the different resources at precisely the same point in time. It is also possible that specific medication interactions classified as moderate/major in some resources may be classified as minor in other resources, excluding specific interactions from our sample size.

We have noted that data collection was convenient for all the subscription resources we used in our study. The information provided was simple, easy to perceive, and located under identifiable categories compared to freely accessible online databases. In the case of free online databases, the information provided was structured in a paragraph where the contents are not tailored to the need or convenient to healthcare professionals.

## Conclusions

The study showed inconsistency among the resources related to information on drug-drug interactions and the number of interacting drug pairs. The study also observed discrepancy for mechanisms, clinical effects, severity, course of action, and level of documentation of the drug interactions among the resources suggesting the need for referring to multiple resources for information related to drug interactions. Significant caution should be exercised while prescribing high alert medications, drugs with a narrow therapeutic index, and drugs metabolized by cytochrome P450 that can lead to clinically relevant unpredictable drug interactions, especially in patients with pre-existing medical conditions and advanced age groups. An updated, unbiased, precise information among the DI resources is necessary for the healthcare professional in ensuring patient safety in clinical practice. Psychiatrists should be vigilant about potential drug-drug interactions while selecting appropriate medications and carefully monitoring psychiatric illness in COVID-19 patients.
